# Preclinical studies reveal MLN4924 is a promising new retinoblastoma therapy

**DOI:** 10.1038/s41420-020-0237-8

**Published:** 2020-01-20

**Authors:** Arthur Aubry, Tao Yu, Rod Bremner

**Affiliations:** 1 Lunenfeld Tanenbaum Research Institute, Mount Sinai Hospital, Sinai Health System, 600 University Avenue, Toronto, ON M5G 1X5 Canada; 2 Department of Laboratory Medicine and Pathobiology, University of Toronto, 27 King’s College Circle, Toronto, ON M5S 1A1 Canada; 3 Department of Ophthalmology and Vision Science, University of Toronto, 27 King’s College Circle, Toronto, ON M5S 1A1 Canada

**Keywords:** Eye cancer, Targeted therapies

## Abstract

*RB1* loss (*RB1*^*null*^*)* or *MYCN* amplification (*MYCN*^*amp*^) in fetal human retina causes retinoblastoma. SKP2 loss kills *RB1*^*null*^ cells, but small molecule SKP2 inhibitors remain unexplored therapeutically. Whether SKP2 is synthetic lethal in *MYCN*^*amp*^ retinoblastoma is unclear. SKP2 is the substrate recognition component of two Cullin-RING Ligase complexes (CRL1^SKP2^/SCF^SKP2^, and CRL4^SKP2^), a family of multiprotein E3 ubiquitin ligases. NEDD8 activating enzyme (NAE) is required for Cullin neddylation and thus CRL activation. Here, we show that the NAE inhibitor, Pevonedistat (MLN4924), potently inhibits *RB1*^*null*^ and *MYCN*^*amp*^ tumors. Intravitreal MLN4924 suppressed multiple human xenografts with EC80s from 20 ng to 3.5 μg. Maximum tolerated dose (MTD) was 10–30 μg, highlighting a favorable therapeutic window. Inhibition of Cullin neddylation was similar in all cases, but cellular effects ranged from G1 arrest with apoptosis to G2/M arrest with endoreplication. However, even in less sensitive lines (EC50 ≈ 1 μM), prolonged exposure was lethal or induced persistent cytostasis. Mechanistically, depleting any single Cullin did not fully recapitulate drug phenotypes, but sensitivity to SKP2 loss correlated with that of drug. Thus, intravitreal MLN4924 is a promising new retinoblastoma therapy, mimicking the cancer-specific lethality of eliminating SKP2 complexes.

## Introduction

Retinoblastoma (RB) is an aggressive intraocular childhood cancer that is lethal if not treated. Although most patients survive non-metastatic disease, clinical challenges involve salvaging the globe, retaining vision, and reducing chemotherapeutic toxicity^1^. New therapies are needed to improve safety and efficacy. Intravitreal (IVT) chemotherapy is now a viable modality because of rigorous procedures to prevent tumor spread^2,3^. Local drug administration avoids systemic toxicity, but eye toxicity has been reported with current agents^2,4^, indicating a pressing need for alternatives. Most RB tumors arise through homozygous *RB1* tumor suppressor gene inactivation^5^. However, in addition to *RB1*^null^ cases, some tumors retain *RB1*, but amplify *MYCN*^6^. The extent to which these “*MYCN*^amp^” tumors utilize unique molecular networks and/or require distinct therapeutics is unclear.

Cullin RING ligases (CRLs) regulate the ubiquitylation and turnover of many eukaryotic proteins^7^. They have four components: one of eight Cullin proteins (CUL1, 2, 3, 4A, 4B, 5, 7, or 9) that are scaffolds, one of two ring finger proteins (RBX1 or 2) that recruit the ubiquitin-charged E2 protein; one of four adapters (SKP1, elongin B/C, BTB protein, or DDB1); and many substrate recognition proteins that form hundreds of CRL complexes^7^. CRL1 complexes utilize CUL1, of which a subset are the SCF (**S**KP1-**C**UL1-**F**box) complexes. They utilize the E2 protein and RBX1 on one side of CUL1 to donate ubiquitin to a substrate binding protein pair on the other side (SKP1 bound to one of ~70 F-box proteins). SKP2 is the substrate recognition F-box protein in SCF^SKP2^, and is the E3 ligase that ubiquitylates substrates^8–10^. SKP2 also binds the CUL4A-DDB1 complex which, like SCF^SKP2^, ubiquitylates the CDK inhibitor p27^11^. In recent years, SCF complexes have attracted attention as potential therapeutic targets in cancer^10^. Notably, SKP2 is essential for *RB1*^*−/−*^ cell survival, and its loss in RB upregulates p27 and impairs tumor cell survival in vitro^12^. Whether SKP2 loss is also synthetic lethal in *RB1*^*+/+*^ *MYCN*^amp^ RB tumors is unknown.

Some direct SKP2 inhibitors have been developed, but potency is low, and none are in clinical trials. All CRLs require Cullin neddylation, in which the ubiquitin-like neural precursor cell-expressed, developmental downregulated 8 (NEDD8) peptide is covalently linked to substrates. It involves a cascade similar to the ubiquitin pathway with E1, E2, E3, and deneddylating enzymes^13^. The small molecule MLN4924 (Pevonedistat), an adenosine sulfamate analog, potently and selectively inhibits NEDD8 activating enzyme (NAE), the E1 enzyme^14^. MLN4924 inhibits several solid and liquid cancers in vitro and in vivo^14–18^, and Phase 1 clinical trials demonstrated good systemic tolerance and some efficacy^19–21^. NAE is a dimer of NAE1 and ubiquitin-like modifier activating enzyme 3 (UBA3). It carries out three steps that precede Neddylation of the downstream E2 enzyme UBC12^22^. NAE first generates an AMP-NEDD8 adduct from ATP and NEDD8, which occupies the nucleotide binding pocket. NEDD8 is then transferred to a thiol group, releasing AMP, and lastly, a second AMP-NEDD8 adduct enters the nucleotide binding pocket. This dual NEDD8 bound form of NAE is competent to pass one NEDD8 peptide to UBC12. MLN4924 disrupts the latter step by binding to the nucleotide pocket, using thioester bound NEDD8 to form an MLN4924-NEDD8 adduct, blocking the downstream cascade. Neddylation of substrates like CRLs is inhibited within hours^14,15^. Several other proteins are neddylated such as ribosomal proteins, p53, E2F1, MDM2, VHL, and SMURF1^23^. Consistent with this growing list, as well as the eight CRLs and their myriad targets, MLN4924 has multiple effects including cell cycle arrest, apoptosis, autophagy, senescence, and/or endoreplication^14,15,24,25^. Thus, whether MLN4924 might act principally through SKP2 is unclear.

Here, we show that MLN4924 potently inhibits RB growth in vivo without toxicity at effective doses. The drug inhibits both *RB*^*null*^ and *MYCN*^*amp*^ cells, and depleting SKP2 mimics several effects of the drug on cell cycle and survival. Thus, MLN4924 is a potential new IVT therapy for RB that harnesses the exquisite SKP2-dependency of this pediatric cancer.

## Results

### MLN4924 inhibits RB cell growth in vitro in a time and dose-dependent manner

SKP2 is oncogenic in RB^12^, therefore it is a potential therapeutic target. We compared the efficacy of MLN4024 to Compound A, which blocks SKP2 binding to SCF^26^. We observed time and dose dependent sensitivities for both drugs, but MLN4924 was 14× more potent (EC50_MLN4924_ ≈1 μM vs. EC50_CmpdA_ = 14 μM at 72 h) (Supplementary Fig. 1a–c). MLN4924 is in clinical trials, with promising efficacy^23^, thus we focussed on its effects in preclinical models. MLN4924 inhibited 3D growth in soft agar, further justifying in vivo tests (Supplementary Fig. 1d).

### Intravitreal MLN4924 impedes RB growth in vivo

**Fig. 1:**
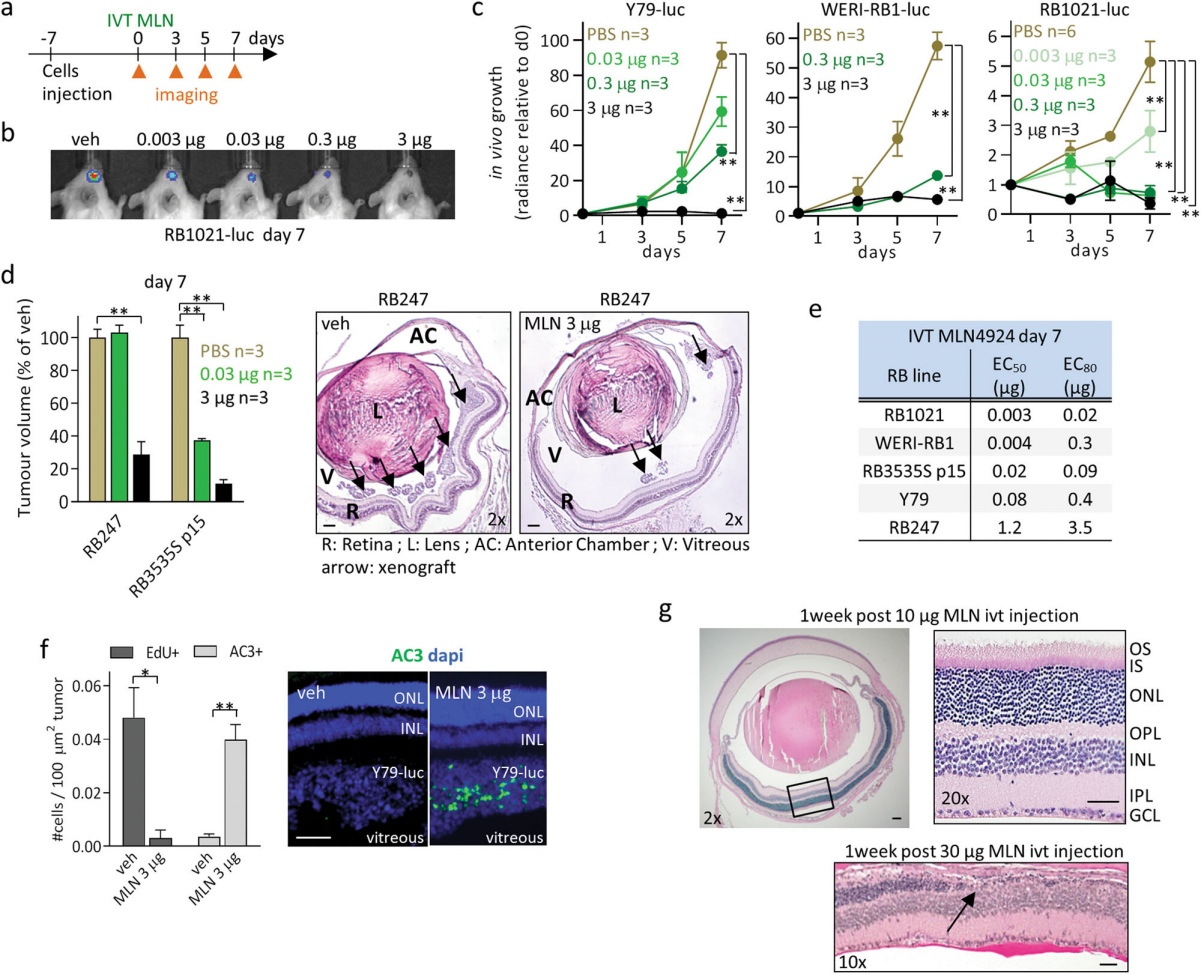
IVT MLN4924 impedes RB growth in vivo. **a** Experimental design for assessing the efficacy of IVT MLN4924 in orthotopic RB xenografts. **b** Representative image of the radiance total flux signal (photons/second) of RB1021-luc tumors treated with the indicated doses of IVT MLN4924 at day 7. **c** In vivo growth curves of Y79-luc, WERI-RB1-luc, and RB1021-luc tumors treated with the indicated doses of IVT MLN4924. Radiance total flux (photons/second) values were acquired at day 0, 3, 5, 7 and normalized to d0 for each tumor, then plotted as mean +/− SD (**P < 0.01 by Student *t*-test). *n* represents the number of mice; tumors were established in the right eye of each animal. **d** On the left—Normalized tumor volumes at day 7 of unlabeled RB247 and RB3535S (p15) treated with the indicated doses of IVT MLN4924; volumes were quantified by planimetric method, averaged, and plotted as percentage +/− SD of PBS vehicle (**P < 0.01 by Student *t*-test); On the right—representative H&E-stained eye section with RB247 tumors and treated as indicated at day 7. Scale bar is 100 μm. **e** Summary of the in vivo EC50s and EC80s of IVT MLN4924 in orthotopic RB xenografts. **f** On the left—quantification of replicating (EdU+) and apoptotic (AC3+) cells in Y79-luc tumors treated as indicated at day 7. Plot shows mean +/− SD (*n* = 3 *P < 0.05, **P < 0.01 by Student *t*-test); On the right—representative AC3 immunofluorescence staining with DAPI of Y79-luc tumors treated as indicated at day 7. Scale bar is 100 μm. **g** Representative eye sections treated as indicated and stained with H&E at day 7. The black arrow pinpoints area of photoreceptor layer disappearance. Scale bars are 100 μm.

Three RB lines (Y79, WERI-RB1, and RB1021) were modified to express luciferase, then 50,000 cells were injected into NOD-Scid vitreous, followed vehicle or drug 7 days later. Live imaging revealed potent dose and time-dependent reduction in tumor growth (Fig. 1a–c). End-point analysis of H&E sections of two unlabeled RB lines (RB247, RB3535S low passage p15) also revealed dose-dependent inhibition (Fig. 1d). EC50 ranged from 3 to 1200 ng and EC80 from 20 to 3500 ng (Fig. 1e). Drug treated-tumors had less dividing and more apoptotic cells (Fig. 1f). Histological examination revealed no toxicity from 3 or 10 µg doses, but photoreceptor loss was detected at 30 µg (Fig. 1g), indicating the maximum tolerated dose (MTD) in murine vitreous is 10–30 µg, well above the therapeutic dose. Thus, IVT MLN4924 is a promising new therapeutic strategy.

### *RB1*^*null*^ and *MYCN*^*amp*^ cells are sensitive to MLN4924

**Fig. 2:**
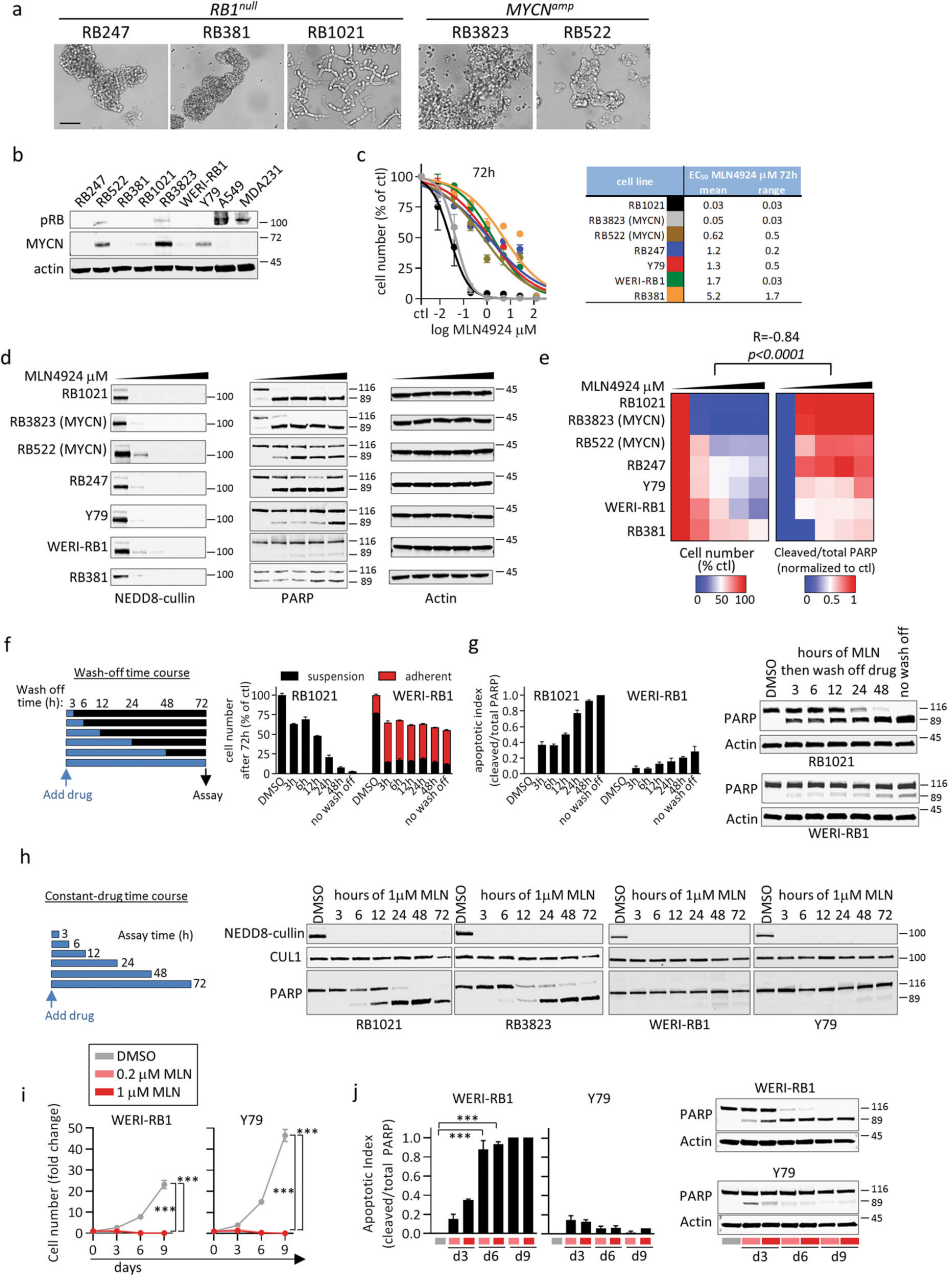
Cellular and molecular effects of MLN4924 on RB cell lines. **a** Representative bright field image of the indicated human *RB1*^*null*^ and *MYCN*^*amp*^ RB cell lines in culture used in this study. Scale bar is 50 μm. **b** Expression profiles of pRB and MYCN were confirmed by Western blot in *RB1*^*null*^ and *MYCN*^*amp*^ RB cell lines. The non-small cell lung cancer A549 and breast MDA-MB-231 cell lines were used as positive control for pRB expression. **c** RB cells (color coded as indicated in the table) were treated with different concentrations of MLN4924 (0, 0.008, 0.04, 0.2, 1, 5, 25, 125 µM) for 72 h, then cell number was quantified using the CellTiterGlo luminescent assay and the data was normalized as percentage of control. The average of two independent experiments is plotted. Error bars indicate the range. In vitro EC50s of MLN4924 and their respective range in RB lines were computed using Graphpad Prism software and summarized. **d**, **e** The indicated RB cell lines were treated with DMSO, 0.2, 1, 5, and 25 μM MLN4924 for 72 h, then protein lysates were prepared to assess levels of Cullin neddylation (NEDD8-Cullin) and apoptosis (PARP cleavage) by Western blot in **d**. In parallel, live cells were counted using trypan blue exclusion. PARP cleavage and cell number data were then normalized to DMSO control in each line. The averages of two biological replicates are shown as heatmaps in **e**. **f**, **g** RB1021 and WERI-RB1 cells were treated with 1 µM MLN4924, and at different timepoints (3, 6, 12, 24, 48 h) the drug was washed-off and replaced by fresh drug-free medium as indicated. At 72 h, floating and adherent live cells were counted and normalized as percentage of control (Supplementary Fig. 2 shows images of the adhesion phenotype). The average of two biological replicates is plotted in **f**. Error bars indicate the range. In parallel, protein lysates were prepared to assess PARP cleavage by Western blot. Quantification (*n* = 2 average +/− range) and a representative blot are shown in **g**. **h** Two hypersensitive (RB1021 and RB3823) and two sensitive (WERI-RB1 and Y79) RB lines were treated with 1 µM MLN4924, and at the indicated timepoints (3, 6, 12, 24, 48, 72 h) protein lysates were prepared to assess levels of Cullin neddylation (NEDD8-Cullin) and apoptosis (PARP cleavage) by Western blot. **i**, **j** Less sensitive WERI-RB1 and Y79 cells were treated with 0.2 and 1 µM MLN4924 for 9 days. Cell number was quantified as previously at d3, d6, d9, and normalized to d0. In parallel, apoptosis was quantified by PARP cleavage. Cell growth and apoptosis data are graphed in **i** and **j**, respectively (*n* = 3 mean +/− SD ***P < 0.001 by Student *t*-test).

To broaden utility and examine mechanism, we tested five *RB1*^*null*^ and two *MYCN*^*amp*^ RB cell lines in vitro. All grew as semi-adherent clusters or chains of spherical cells, typical of RB lines (five examples in Fig. 2a). Westerns confirmed that *RB1*^*null*^ lines (RB247, RB381, RB1021, WERI-RB1, Y79) lacked pRB, whereas *MYCN*^*amp*^ lines (RB522, RB3823) retained pRB but had high MYCN expression (Fig. 2b), consistent with gene amplification^6^. MYCN is also amplified in *RB1*^*null*^ Y79 cells^27^, where its expression was also high, while MYCN was detected at lower levels in RB1021 and WERI-RB1 cells (Fig. 2b).

All seven lines were sensitive to MLN4924, with EC50s in the clinically relevant nM to low μM range (Fig. 2c). RB381 was least sensitive (EC50 ≈ 5 µM), 4/7 lines (RB522, RB247, Y79, and WERI-RB1) had an EC50 ≈ 1 µM, and 2/7 (RB1021 and RB3823) were hypersensitive with EC50s of 30–50 nM (Fig. 2c). These differences did not correlate with *RB1* or *MYCN* status (Fisher exact test, *P* = 1.0). Cullin neddylation was ~complete across all cell lines and doses (Fig. 2d) but PARP-cleavage, which marks Caspase-driven apoptosis, was evident in all lines but more prominent at lower doses in hypersensitive lines (*R* = 0.84, *P* < 0.0001, Fig. 2d, e).

To define the minimal period of exposure required for apoptosis we performed time-course assays, using both wash-off and constant-drug strategies, and in both hypersensitive and sensitive lines (Fig. 2f–h). For wash-off assays, media with 1 µM MLN4924 (EC95/RB1021, EC50/WERI-RB1 at 72 h) was replaced with drug-free media at various times and cell number and apoptosis assessed at 72h (Fig. 2f, g). Three hours (h) exposure was sufficient to provoke a comparable growth defect in both hypersensitive RB1021 and less sensitive WERI-RB1 cells (Fig. 2f). In RB1021 cells PARP cleavage rose from ~40% to ~100% with 3 or 72 h drug treatment, respectively, and from <5 to ~20% in WERI-RB1 cells (Fig. 2g). Thus, short exposure to the same dose of MLN4924 is sufficient to induce considerable or partial apoptosis.

For constant-drug assays, cells were continuously treated with 1 µM MLN4924 and harvested at various times (Fig. 2h). Two hypersensitive RB lines (RB1021/*RB1*^*null*^, RB3823/*MYC*^*amp*^) and 2 less sensitive line (WERI-RB1, Y79; both *RB1*^*null*^) were compared. MLN4924 blocked neddylation in all cases by 3 h. In line with the wash-off assay, PARP cleavage exceeded 50% after 24 h of continuous exposure in hypersensitive lines, whereas less and later cleavage was evident in less sensitive lines (Fig. 2h). These differences are consistent with the dose-response results (Fig. 2c).

In addition to affecting cell number, 3 h exposure to MLN4924 also committed WERI-RB1 to a persistent adherent, flattened phenotype, accompanied with spiked filopodia-like protrusions and instances of multinucleated cells (quantification of adhesion is shown in Fig. 2f, a representative image of the adhesion phenotype is shown in Supplementary Fig. 2a). RB522 cells exhibited a similar response, although far fewer cells were affected (data not shown). MLN4924 induces senescence in some contexts^23^, but unlike etoposide-treated A549 lung cancer cells, MLN4924-treated WERI-RB1 cells lacked senescence-associated β-galactosidase (SA-βgal) (Supplementary Fig. 2b). Instead, flattened cells showed strong phalloidin staining in filopodia-like protrusions (Supplementary Fig. 2a). We also noted phalloidin-positive protrusions in MLN4924-treated RB1021 cells, but the cells did not adhere (Supplementary Fig. 2a). Using cyclic arginine-glycine-glutamate (cRGD) peptides as integrin decoys to disrupt adhesion, either concomitant (MLN4924 + cRGD) or sequential (MLN4924 → cRGD) treatment efficiently disrupted adhesion, but did not alter PARP cleavage or cell number (Supplementary Fig. 2c–e). AnnexinV/DAPI staining followed by flow cytometry confirmed apoptosis was unaffected after 72 h exposure (Supplementary Fig. 2f). Thus, MLN4924 induces reversible integrin-mediated adhesion, not senescence, in WERI-RB1 cells, which is uncoupled from effects on cell number and cell survival.

The time-dependent effects of MLN4924 (Fig. 2f–h) led us to ask if extended exposure improves efficacy in less sensitive RB lines. Three days exposure to 200 nM or 1 μM MLN4924 eliminated virtually all RB1021 and RB3823 cells, but reduced WERI-RB1 or Y79 cells by approximately 30–40% (Fig. 2c). Extending treatment to 6 or 9 days potently inhibited WERI-RB1 and Y79 growth, and stimulated massive apoptosis in the former by 6 days (Fig. 2i, j). These results highlight the potential of extended exposure.

### Distinct cell cycle effects of MLN4924 in different RB tumors

Reduced cell number without apoptosis implies cell cycle arrest, and apoptosis can follow arrest in any of several cell cycle phases, thus we assessed effects on cell cycle stage. Five RB cell lines were treated with 200 nM MLN4924 for 24, 48, and 72 h, and then pulsed for 30 min with the nucleotide analog EdU prior to fixation, staining, and flow cytometry.

**Fig. 3:**
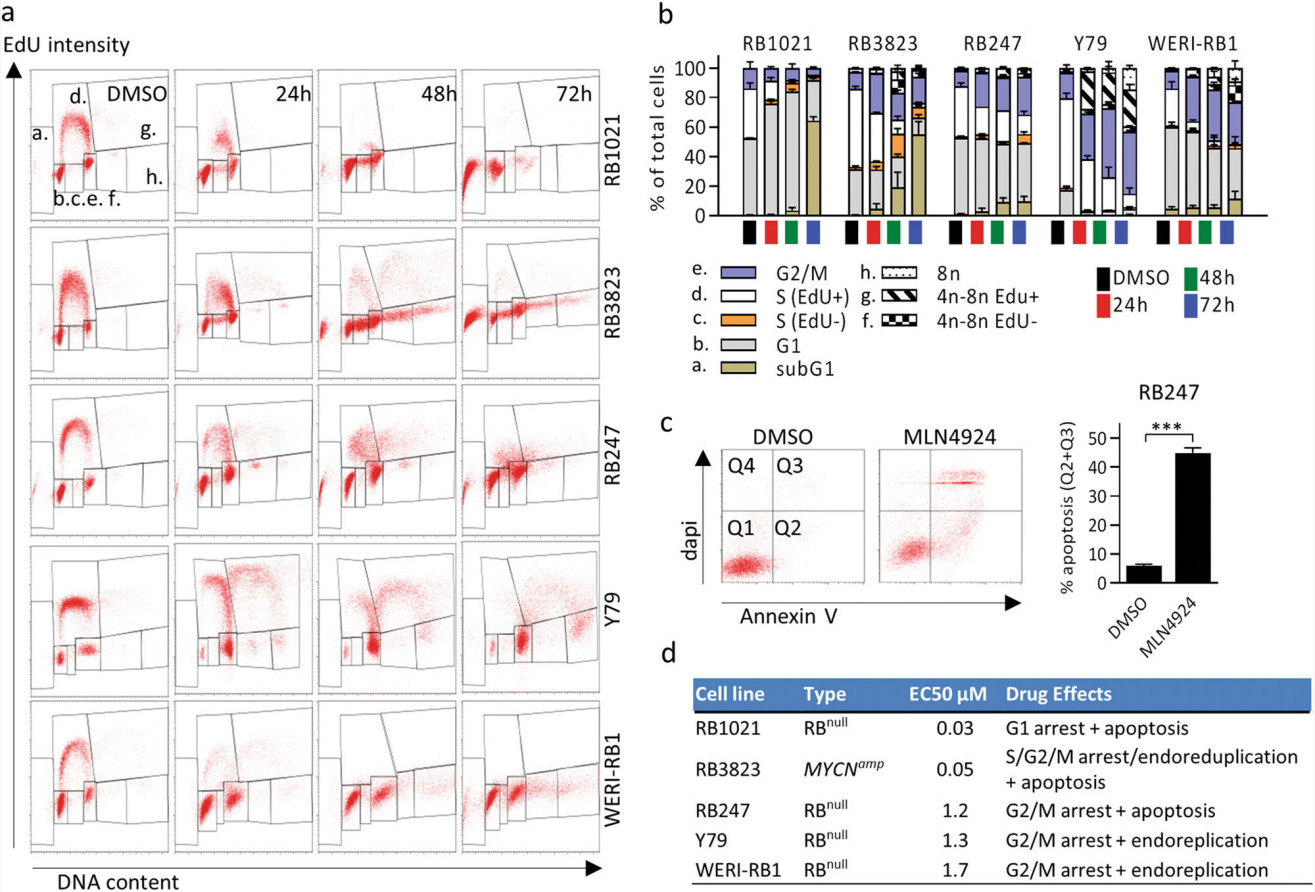
Cell cycle effects of MLN4924 in RB. **a** The indicated 5 RB cell lines were treated with DMSO for 72 h or 200 nM MLN4924 for 24, 48, and 72 h, and at each timepoint the cells were exposed to EdU for 30 min prior to fixation/permeabilization, click chemistry, DAPI staining, and flow cytometry to assess the effect on the cell cycle. A representative set of plots is shown. The gates **a**–**h** represent cell cycle phases as explained in **b**. **b** Data from the assays in **a** was summarized in bar charts. Averages are plotted and error bars indicate the range of 2 assays. **c** Apoptosis of RB247 cells was confirmed by comparing Annexin V/dapi staining in vehicle (DMSO) vs. MLN4924 (200 nM) treated cells. A representative flow cytometry plot is shown and quantification is also graphed (*n* = 3 average +/− SD, ***P < 0.0001 by Student *t*-test). **d** Summary of the effects of MLN4924 on different RB cell lines.

Drug responses were distinct in different RB lines. In agreement with PARP data (Fig. 2d, e), flow cytometry confirmed that both hypersensitive lines (RB1021 & RB3823: EC50 30–50 nM) underwent considerable apoptosis (>50% subG1 cells; Fig. 3a, b). However, death was preceded by G1 arrest in RB1021 cells, but S + G2/M arrest and endoreduplication in RB3823 cells. The three less sensitive lines (RB247, WERI-RB1, Y79: EC50 > 1 µM) all underwent mainly G2/M arrest, but while WERI-RB1 and Y79 re-entered S-phase (endoreplication) and generated polyploid cells, RB427 cells arrested in G2/M without endoreplication (Fig. 3a, b). PARP analysis indicated that RB427 cells underwent apoptosis (Fig. 3d, e), but FACS detected few subG1 cells (Fig. 3a, b). RB247 grows in tight clumps (Fig. 3a) and fixed cells complicate FACS studies, suggesting this difference is technical. Indeed, DAPI penetration and Annexin V analysis confirmed considerable apoptosis in MLN4924 treated RB247 cells (Fig. 3c). In summary, RB cells show various responses to MLN4924, including G1 arrest + apoptosis, S/G2/M arrest + apoptosis, G2/M arrest + apoptosis, or G2/M arrest + endoreduplication (Fig. 3d). This variety likely reflects multiple deneddylated proteins interfacing with variable molecular networks across RB tumors.

### SKP2 depletion partially mimics the effects of MLN4924

MLN4924 inhibits many Cullin-based ubiquitylating complexes that target hundreds of proteins for degradation, and also inhibits neddylation of non-Cullin substrates. A survey of five proteins targeted by various Cullin complexes (p53, p21, p27, p130, and CTD1) suggested that MLN4924 has distinct molecular effects in different RB tumors; for example, p27 was more potently induced in RB3823 *MYCN*^*amp*^ cells versus *RB1*^*null*^ cells, but p21 showed the opposite behavior; p130 was only induced (albeit modestly) in the hypersensitive lines RB1021 and RB3823; and while p53 and CDT1 were induced ubiquitously, the response was earlier in these hypersensitive cells (Supplementary Fig. 3). These data suggest Cullins contribute to the differential effects of MLN4924.

To directly assess Cullin involvement, we asked whether depleting any of five Cullins recapitulates any of the effects of MLN4924. We also depleted SKP2, a component of CUL1 and CUL 4 complexes that is critical for survival of *RB*^*−/−*^ cells^12^. Six cell lines were exposed to control or test siRNA for 6 days, longer than the 3-day drug assay to ensure protein depletion. Westerns confirmed efficient knockdown in all cases (Supplementary Fig. 4a). Interestingly, SKP2 knockdown reduced CUL1 levels in 5/6 lines, and they associate^8,9^ the complex may stabilize CUL1. Also, CUL2 knockdown moderately reduced SKP2 levels, suggesting CUL2 promotes SKP2 stability or expression (Supplementary Fig. 4a). CUL2, 3, 4A, or 4B siRNAs each depleted their target, but without altering other Cullins or SKP2 (Supplementary Fig. 4a).

**Fig. 4:**
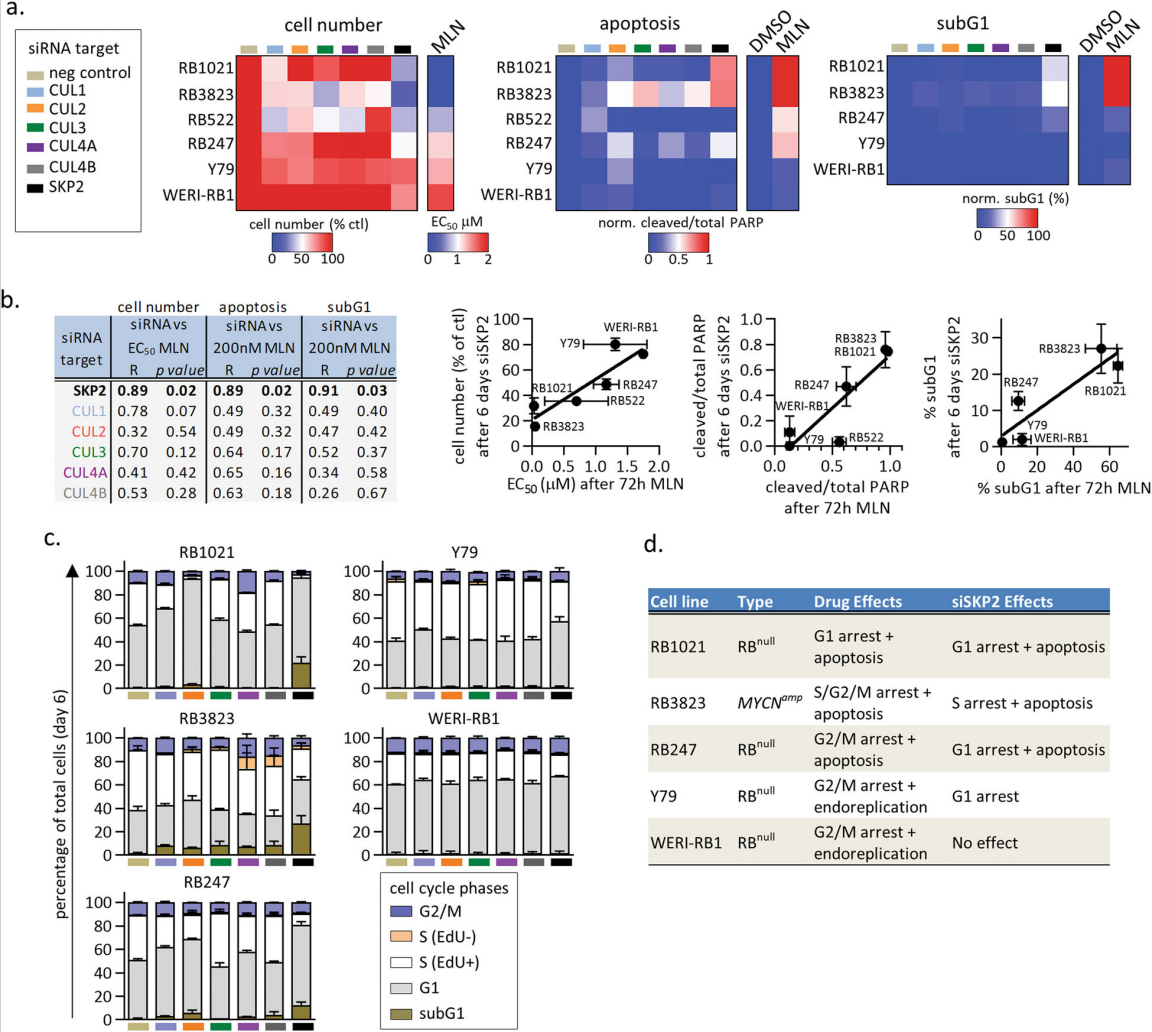
Skp2 depletion mimics many of the effects of MLN4924. **a** The listed siRNAs (color coded as indicated) were used to deplete the corresponding target in the six listed retinoblastoma cell lines (knockdown efficiency shown in Supplementary Fig. 3a). After 6 days, live cell counts were obtained, Westerns were run to quantify PARP cleavage (Supplementary Fig. 3a, b shows an example blot), FACs assays were run to define subG1 cells (see **c** for full cell cycle data, and Supplementary Fig. 3c for examples of the FACS plots), and the results, normalized to siCtrl, were displayed in heat maps (averages of two biological replicates are plotted). **b** Data from each of the siRNA assays in **a** were plotted against similar data obtained with MLN4924 (*n* = 2 average +/− range). The table summarizes the *R* and *p* values for each siRNA tested, and the graphs for siSKP2 are shown on the right. **c** Bar graphs summarizing data from FACS analysis on the five indicated cell lines treated with the indicated siRNAs, color-coded as in **a** (*n* = 2 average +/− range). Examples of the FACS plots are provided in Supplementary Fig. 3c. **d** Summary of the effects of drug *vs*. siSKP2 on cell cycle and survival in five RB lines. The cell lines are ordered most to least drug sensitive from top to bottom, respectively.

We tracked cell number and PARP cleavage in 6/6 of the RNAi treated lines, used FACS to track cell cycle phase in 5/6 lines, and then calculated the correlation between siRNA vs. drug effects in each assay (Fig. 4a–c, Supplementary Fig. 4b, c). Cell death was also confirmed visually (e.g., Supplementary Fig. 4d). The effects of SKP2-depletion on cell number and apoptosis (assessed by either PARP cleavage or subG1 cells), correlated with the effects of MLN4924; lines that were hypersensitive to drug (e.g., RB1021, RB3823) were also the most sensitive to siSKP2, and less drug-sensitive lines (e.g., Y79, WERI-RB1) were least affected (Fig. 4a, b). In contrast, although depleting Cullins affected cell number and survival in some contexts (Fig. 4a, and see below), correlations with drug treatment across all tested lines did not reach significance (Fig. 4b). Consistent with its contribution to the SCF^SKP2^ complex, CUL1 depletion reduced cell number in 5/6 lines (more than loss of any other Cullin), and triggered apoptosis in lines where MLN4924 also had this effect, but the magnitude of the effects did match that of SKP2-depletion. These data suggest that SKP2 inhibition is a major effector of MLN4924 in RB cells, and is likely mediated by both CUL1 and CUL4 complexes that utilize this protein.

RNAi also revealed a unique response in *MYCN*^*amp*^ RB3823 cells. Depleting any of the 5 Cullins reduced cell number and increased cell death, whereas in *RB1*^*null*^ cells only select Cullins had this effect, and with less magnitude (Fig. 4a, b). Heightened responsiveness to disruption of individual Cullins provides a molecular explanation for the exquisite sensitivity of RB3823 cells to MLN4924 (Fig. 1c).

EdU-labeling and flow cytometry provided insight into the cell cycle effects of Cullin or SKP2 depletion. In RB1021, siSKP2 caused G1 arrest and apoptosis, exactly like MLN4924 (Fig. 4c, d, Supplementary Fig. 4c). In RB3823, siSKP2 reduced the fraction of S cells and caused apoptosis, partially mimicking MLN4924. In RB247, there was cell cycle arrest and apoptosis with either siRNA or drug, but while siSKP2 arrested cells in G1 phase, the drug caused G2/M arrest. In Y79 and WERI-RB1, while the drug induced G2/M arrest and endoreplication, siSKP2 caused G1 arrest in Y79 and had no effect in WERI-RB1. Therefore, SKP2-depletion partially mimics the cell cycle effects of MLN4924, especially in hypersensitive lines (Fig. 4d). Other Cullin complexes likely contribute to the cell cycle effects, as there were cell-specific and family member-specific effects of depleting the 5 Cullins (Fig. 4c, Supplementary Fig. 4c). For example, CUL2 loss triggered modest G1 accumulation in RB1021, RB247, and Y79 cells, CUL3 potently induced G1 arrest in RB1021 cells with a more modest effect in RB247 cells, while CUL4A or CUL4B loss triggered S/G2/M arrest in RB3823 cells. As with the cell number and survival effects (Fig. 4a), Cullin depletion was more likely to influence the cell cycle in the most sensitive lines (Fig. 4c, d). Altogether the data paint a consistent picture in which the sensitivity of RB cells to MLN4924 matches their dependency on SKP2 and Cullins.

## Discussion

Here we reveal the therapeutic potential for RB of the small-molecule NAE inhibitor MLN4924. Local IVT delivery demonstrated time-dependent and dose-dependent tumor suppression without toxicity. Drug efficacy in different RB tumors relied on distinct growth-suppressive cellular effects, despite similarly robust inhibition of Cullin neddylation, suggesting various downstream mechanisms of action. Depleting Cullins mimicked some drug effects, but did not correlate with overall drug sensitivity. In contrast, there was a strong correlation between drug efficacy and sensitivity to SKP2 loss, suggesting that MLN4924 cytotoxicity was attributable in part to inhibition of CRLs with SKP2, which includes CRL1^SKP2^/SCF^SKP2^ and CRL4^SKP2^ ^8,9,11^.

We tested two strategies to inhibit SKP2. We challenged RB cell lines with compound A (cpdA), a direct SKP2 inhibitor, and MLN4924, a NAE inhibitor, as an alternative to target SCF^SKP2^. MLN4924 inhibits CRLs because Cullin neddylation is required to prevent inhibitory binding by CAND1^28^. The sensitivity of RB cells to cpdA and MLN4924 were in the same range as other cancers^14,26^, however MLN4924 was 14× more potent. Moreover, because MLN4924, but not cpdA, is in clinical trials for many cancers^19^ we further characterized its potential in vivo.

MLN4924 does not cross the blood brain barrier, but IVT chemotherapy is now standard-of-care in several centers^29^. IVT MLN4924 was remarkably effective against orthotopic RB xenografts, without toxicity, thus it is a promising therapeutic strategy. Drug retention time and extent of diffusion remain to be addressed. In vitro analysis revealed that 3 h exposure is sufficient to reduce growth by 50%, and extending this time improved responsiveness drastically. These observations may help design pharmacokinetic studies for IVT MLN4924.

Many RB therapies induce DNA damage^1^ and MLN4924 synergizes with DNA damaging agents^30^, including radiation^31–33^. Our in vivo study showed IVT MLN4924 was effective, however in the schedule used we did not observe any complete responses (CR), therefore residual tumor cells would likely regrow. RB xenografts are difficult to eradicate, similar to difficult-to-treat vitreal seeds in the human eye^34–37^. Therefore, assessing multiple injections or combining MLN4924 with standard-of-care chemotherapies are attractive strategies to achieve CR. It would also be of interest to test MLN4924 efficacy in metastatic RB or on more aggressive primary tumors that lead to enucleation, which can now be predicted from somatic chromosomal copy-number alterations (SCNA) detected in aqueous humor extracts^38^.

Akin to other cancers^14^, we observed robust inhibition of Cullin neddylation in RB. However, drug responses were distinct in different RB cell lines, suggesting engagement of differential circuitry. Most RB tumors initiate through *RB1* loss, but *MYCN* amplification drives a rare subset^6^. *RB1* or *MYCN* status did not dictate responsiveness as, for example, the *RB1*^*null*^ RB1021 line was more sensitive than two *MYCN*^*amp*^ lines, whereas other *RB*^*null*^ lines were less sensitive. In *MYCN*^*amp*^ RB3823 cells, disrupting any of 5 Cullins or SKP2 compromised survival, consistent with high sensitivity to MLN4924. *MYCN*^*amp*^ tumors are more aggressive than *RB1*^*null*^ cancers, arising months earlier^6^. MLN4924 could, therefore, be valuable to treat this RB subset. The second *MYCN*^*amp*^ cell line (RB522) had a higher EC50, but irrespective, the EC50s of all *RB*^*null*^ and *MYCN*^*amp*^ cell lines were in the clinically relevant range, with a subset being hypersensitive. Our data indicates that, despite distinct genetic origins, both *RB1*^*null*^ and *MY*C*N*^amp^ tumors are SKP2-dependent and exhibit sensitivity to MLN4924.

MLN4924 promoted distinct cell cycle phenotypes in RB and depleting Cullins or SKP2 recapitulated some of these effects. For example, MLN4924 induced G1 arrest in RB1021 cells, as did CUL1 or SKP2 depletion. However, depleting single Cullins was insufficient to recapitulate all the cell cycle effects in most RB lines, suggesting Cullin redundancy and/or engagement of other Neddylated proteins. For example, while MLN4924 induced endoreplication in several RB cell lines (RB3823, Y79, and WERI-RB1), this was not recapitulated by depleting any Cullin. Consistent with that result, Cullins redundantly ubiquitylate the replication licensing factor CDT1, and co-knockdown of CUL1/4A/4B is necessary to mirror MLN4924-induced endoreplication in HCT-116 colon cancer cells^39^. In ovarian cancer, depleting CUL4A mimics MLN4924-induced death, and depleting the replication origin licensing factor CDT1, a CUL4A target, rescues these effects^40^. None of the SKP2 or CUL siRNAs mimicked the MLN4924 induced re-replication phenotype in Y79 and WERI-RB1 cells, suggesting that this effect reflects modulation of multiple CRL targets and/or other neddylated proteins.

MLN4924 targets have been linked with specific CRLs^41^, but the precise relationship between Cullins and drug efficacy has not been widely examined. CUL1 RNAi somewhat recapitulated SKP2 RNAi and MLN4924 sensitivity, suggesting that SKP2 is a central effector of MLN4924 in RB, at least in part through the activity of SCF^SKP2^. Analysis of independent large-scale RNAi and CRISPR datasets (Achilles, Colt2, and DRIVE) show a synthetic lethal effect of SKP2 inhibition in *RB1* null cancers^42^. Together, this suggests that using MLN4924 to inhibit SKP2 could be effective in other *RB1* deficient cancers.

In conclusion, we have characterized the in vivo efficacy, toxicity, and the cellular and molecular effects of MLN4924 in RB. Preclinical data in multiple RB xenografts revealed efficacy and safety. Both *MYCN*^*amp*^ and *RB1*^*null*^ cells responded to MLN4924, but there were distinct effects in different RB tumors. Sensitivity to MLN4924 was linked to apoptosis, various forms of cell cycle arrest, and SKP2 inhibition. Thus, MLN4924 is a promising approach to exploit the SKP2-dependency of *RB1* null cancers.

## Methods

### Compounds and cell culture

MLN4924 and compound A were initially gifts from the Ontario Institute for Cancer Research drug library (Toronto, ON, Canada), then MLN4924 was purchased from Vibrant Pharma Inc. (Brantford, ON, Canada). MLN4924 was resuspended in DMSO or sterile phosphate buffer saline (PBS) for in vitro and in vivo work, respectively. Insoluble MLN4924 was sonicated to homogenize drug before in vivo delivery. The RB cell lines RB1021, RB3823, RB381, RB522, RB247, and the low passage RB3535S were grown in T25 flasks in Iscove’s media supplemented with 0.0004% (v/v) β-mercaptoethanol, and 10 mg/L insulin as described^43^. WERI-RB1 and Y79 cells were cultured in RPMI1640, and A549 and MDA-MB-231 cells in DMEM.

### Efficacy in orthotopic xenograft models

Animal protocols were in accordance with local and national guidelines. All animals were handled in agreement with the standard operating procedures of the University Health Network and all procedures were approved by the Animal Care Committee of the University Health Network, Toronto. IVT injections were performed under general anesthesia using isoflurane. Human RB cell lines were prepared at 25,000 cells/μL in sterile PBS with 10% matrigel (BD Bioscience, Mississauga, ON, Canada) and 5% trypan blue, then 2 μL of the mixture were injected into the right vitreous of 3–4 weeks-old NOD-Scid mice. Uninjected left eye served as a negative control. We used three animals per dose tested (unless specified otherwise). After seven days, MLN4924 or PBS was injected in tumor-bearing eyes at the indicated doses. Luciferase^+^ tumor cells were tracked live by i.p injection of d-luciferin at 150 mg/kg for 10 min and radiance total flux (photons/second) tracked with the Xenogen IVIS Imaging System 100 (PerkinElmer, Woodbridge, ON, Canada). For other RB lines, tumor volume was evaluated on sections as described^44^. A human marker (intact mitochondria antibody, MAB1273, Millipore, Etobicoke, ON, Canada) was used to delineate tumor cells.

### Eye toxicity

MLN4924 was injected in the vitreous of anesthetized 3–4 weeks-old NOD-Scid mice at 0.03, 0.3, 3, 10, 30 µg, and 7 days later mice were sacrificed, the eyes enucleated and incubated in Davidson’s fixative overnight at 4 °C on a shaker. Ethanol dehydration and paraffinization were done with a tissue processor (Excelsior ES, Thermo Scientific, Burlington, ON, Canada). Sections (5 µm) were prepared using an ultramicrotome (Leica Microsystems, Richmond Hill, ON, Canada). Sections were deparaffinised and rehydrated before staining with hematoxylin and eosin (H&E).

### Cell growth assays

#### 2D culture; 96-well format

RB cells were seeded at 10,000 cells/100 µl/well. Drugs were prepared by serial dilutions at 6x concentrations, and 20 µl added to cells. Viability was assessed at 0, 24, 48, and 72 h with CellTiter-Glo Reagent (Promega, Madison, WI, USA) and luminescence quantified with a plate reader (EnVision, Perkin Elmer, Woodbridge, ON, Canada). *6-well format:* RB cells were seeded at 700,000 cells/2ml. The next day (day 0 of the assay), MLN4924 was prepared at 5× and 0.5 ml added to cells. Cell counts were performed with trypan blue.

#### 3D culture 96-well format

Each well was first plated with 50 μl of medium containing 0.6% agar, then Y79 cells were seeded at 600 cells/60 μl medium containing 0.35% agar. Drugs were prepared at 2.8× and 60 μl added on top of the cell layer. After 6 days, colonies were assessed with Alamarblue Cell Viability Reagent (ThermoFisher, Burlington, ON, Canada) and absorbance quantified with a plate reader as above.

### Cell adhesion assay

Cells were treated with either MLN4924, cRGD peptide, or both concomitantly or sequentially. At various times, plates were flicked gently to test adhesion. Medium was collected, and adherent cells collected after trypsinization. Cells in suspension and adhesion fractions were counted with trypan blue.

### Gene silencing

RB cells were seeded in 6-well plates at 700,000 cells/2ml medium with 10% FBS. siRNA mixes with DharmaFECT 1 Transfection Reagent (Horizon Discovery, Denver, Lafayette, CO, USA) were prepared as per the manufacturer’s instructions at a final concentration of 50 nM. Mixes were added at day 0 and day 3, and cells were passed if >80%. Confluent. Cell count, western blots, and cell cycle analyses were done at day 6. siRNAs were from Qiagen (Toronto, ON, Canada): negative control SI03650318, CUL1 SI00053417, SKP2 SI00287819, and from Dharmacon siGENOME (Horizon Discovery, Denver, Lafayette, CO, USA): CUL2 D-007277-02, CUL3 D-010224-05, CUL4A D-012610-02, CUL4B D-017965-01.

### Western blotting

Cells were lysed for 1 h on ice in RIPA buffer (Santa Cruz, Mississauga, ON, Canada) supplemented with protease inhibitor cocktail, sodium orthovanadate and PMSF. Lysates were run on 4–20% SDS-PAGE gradient gels, transferred to nitrocellulose, and analyzed by Li-Cor system (LI-COR Biosciences, Lincoln, NE, USA) with antibodies listed in Supplementary Table 1.

### Cell cycle EdU/DNA staining

Cells in 6-well plates were labeled using the Click-iT EdU Alexa Fluor 647 Flow Cytometry Assay Kit then counterstained for DNA with FxCycle Violet dye (ThermoFisher, Burlington, ON, Canada). At least 10,000 single cells per sample were acquired using the Gallios flow cytometer (Beckman Coulter, Mississauga, ON, Canada) and analysed with Kaluza software.

### Fluorescence microscopy

#### Cultured cells

In 6 well plates, glass coverslips were pre-coated with 50 μg/ml of poly-d-lysine and 700,000 cells were seeded in 2 ml/well overnight. At the indicated times, cells were fixed with 4% paraformaldehyde for 10–15 min, permeabilized and blocked for 1 h, then probed with primary antibodies overnight at 4 °C as in Supplementary Table 1. After 2–3 washes, cells were probed with Alexa Fluor 488-conjugated or 568-conjugated secondary antibodies (ThermoFisher, Burlington, ON, Canada) and 4,6-diamidino-2-phenylindole (DAPI) or propidium iodide (PI) for 90 min Coverslips were mounted using VectaShield (Vector Laboratories, Burlington, ON, Canada). High resolution confocal images were acquired with the Wave FX Spinning Disc Confocal microscope (Quorum Technologies, Puslinch, ON) and Volocity software.

#### Cryosections

Mice were i.p. injected with 10 mg/kg EdU for 1 h before sacrifice. Frozen eye sections were blocked and permeabilized in PBS with 5% donkey serum and 0.1% Tween20 for 1 h 30 at R.T. Slides were incubated with the click reaction mix, and antibody staining performed as described^45^. Dilution and buffer for staining with the cleaved caspase 3 (AC3) antibody is indicated in Supplementary Table 1. One in every eight sections throughout the eyes were quantified for AC3+ or EdU+ cells in tumors, and normalized per area of tumor delineated as described above.

### Annexin V and FxCycle violet staining

Cells were washed with PBS and resuspended in 150 µl staining buffer with alexa Fluor® 488 annexin V (ThermoFisher, Burlington, ON, Canada) and 1.5 µg/ml FxCycle Violet dye for 25 m at RT. At least 10,000 single cells per sample were acquired using flow cytometry as above.

### Bright field images

Sections stained with H&E and fixed cells stained with SA β-galactosidase were captured with the Olympus BX61 microscope, and cultured cells were captured with the Zeiss Axio Vert.A1 microscope.

### Senescence-associated β-galactosidase staining

In 6-well plates, cells were seeded on poly-d-lysine pre-coated coverslips as described above, and after drug treatment the cells were fixed and stained using SA-*β-*galactosidase staining kit (Cell signaling, Danvers, MA, USA).

### Statistical analysis

GraphPad Prism statistical software (San Diego, CA, USA) was used to calculate EC50s, *t*-test and related two-tailed *P*-values, as well as Pearson correlation coefficients and related *P*-values.

## Supplementary information

Supplementary Figure Legends

Supplementary Figure 1

Supplementary Figure 2

Supplementary Figure 3

Supplementary Figure 4

Supplementary table 1

authors contribution form
