# Supplementary material for: Preclinical studies reveal MLN4924 is a promising new retinoblastoma therapy
Source: Cell Death Discov. 2020 Jan 20;6:2. doi: 10.1038/s41420-020-0237-8 (PMC7026052; doi:10.1038/s41420-020-0237-8)
Supplement: Supplementary file 1 — Supplementary Figure Legends [file 41420_2020_237_MOESM1_ESM.docx]

**SUPPLEMENTARY FIGURE LEGENDS**

**Supplementary Fig. 1: RB cell lines are sensitive to MLN4924 in a time- and dose-dependent manner**

**a.** 2D growth curves of WERI-RB1 and Y79 treated with the indicated concentrations of Compound A. Cell number was quantified at the indicated timepoints with the CellTiterGlo Luminescent assay and normalized to DMSO at 1h. Plots show the average of two independent assays, and the error bars indicate range.

**b.** The same growth curves as in (**a**) were performed with MLN4924.

**c.** 2D growth inhibition curves of Compound A and MLN4924 at 72h in WERI-RB1 and Y79. The 72h data obtained in (**a**) and (**b**) for each line were normalized to DMSO and graphed. The EC50s with their 95% confidence interval (CI) are summarized.

**d.** 3D growth inhibition curve of MLN4924. Y79 colonies were grown in soft agar for 6 days, quantified with the AlamarBlue dye and normalized to DMSO. Plot shows the average of two independent assays, and the error bars indicate range.

**Supplementary Fig. 2: MLN4924 induces integrin-mediated adhesion in WERI-RB1 cells**

**a.** Representative confocal images of the indicated cell lines stained with phalloidin were captured to assess cell adhesion and changes in the actin cytoskeleton after 24h of 200 nM MLN4924. Propidium iodide was used to label nuclei. WERI-RB1 images show a single confocal plan, and RB1021’s show 3D modeling from z stacks. Yellow arrows pinpoint filopodia-like protrusions. Magnification is 120x for all images. Scale bar is 15 µm.

**b.** Representative bright field images of WERI-RB1 treated 48h with DMSO or 200 nM MLN4924 and labeled for SA-β-galactosidase activity. A549 cells treated 4 days with 1 µM etoposide were stained in parallel and used as positive control. Magnification is 10x for all images. Scale bar is 30 µm.

**c.** Representative bright field images of WERI-RB1 treated as indicated. Magnification is 10x. Scale bar is 30 µm.

**d.** Protein samples at 72h were prepared from the same experiment in (**c**) and Western blot were run to assess apoptosis by detecting PARP cleavage.

**e.** Quantification of live cells in suspension vs adherent after 24 and 72h of the experiment in (**c**). Bar chart shows mean +/- range of two independent assays

**f**. Quantification of the proportion of annexin V + dapi positive cells at 72h in the experiment in (**c**) by flow cytometry. Bar chart shows mean +/- range of two independent assays. An example of flow plot is shown.

**Supplementary Fig. 3: Molecular Effects of MLN4924 on various Cullin target proteins**

**a**. Western blots were run to assess the effect of 1 µM MLN4924 on five Cullin targets in the indicated cell lines after the indicated drug exposure time. Phosphohistone H3 (PH3) is a mitotic marker, and confirms the cell cycle arrest in each line.

**b**. Fold induction after normalization to actin is plotted. n = 2, error bars represent the range.

**Supplementary Fig. 4: Effect of depleting Cullins or SKP2 on RB cells**.

**a**. The listed siRNAs (color coded as indicated) were used to deplete the corresponding target in 6 RB cell lines. Western blots were run to confirm knockdown efficiency. The heatmap represents quantification of the Western data. Arrows highlight efficient knockdown of the target protein. Asterisks indicate cross-regulation of the indicated protein if it occurred in multiple cell lines.

**b.** After 6 days of siRNA treatment Westerns were run to define the extent of PARP cleavage, normalized to Actin. Quantification of the PARP data is shown in Fig 4a & b.

**c.** Cells were pulsed with EdU then flow cytometry was used to define the fraction of cells in different phases of the cell cycle. Quantification of the data is shown in Fig 4a & c.

**d.** Example of apoptotic RB1021 cells (yellow arrows) treated with the indicated siRNA. Scale bar is 20 µm.
