# Supplementary figures and images for: Preclinical studies reveal MLN4924 is a promising new retinoblastoma therapy
Source: Cell Death Discov. 2020 Jan 20;6:2. doi: 10.1038/s41420-020-0237-8 (PMC7026052; doi:10.1038/s41420-020-0237-8)

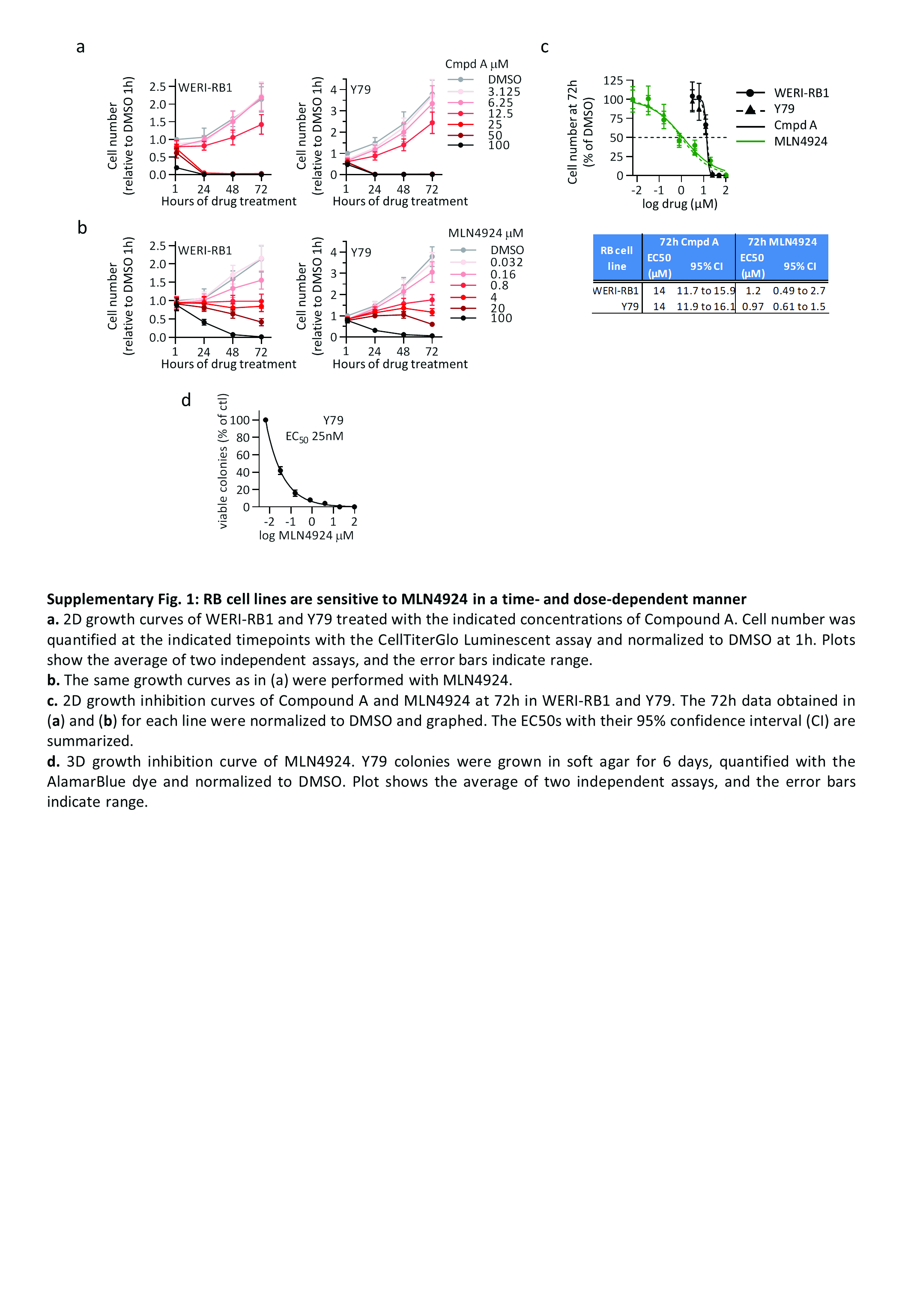

Supplement: Supplementary file 2 — Supplementary Figure 1 [file 41420_2020_237_MOESM2_ESM.tif]

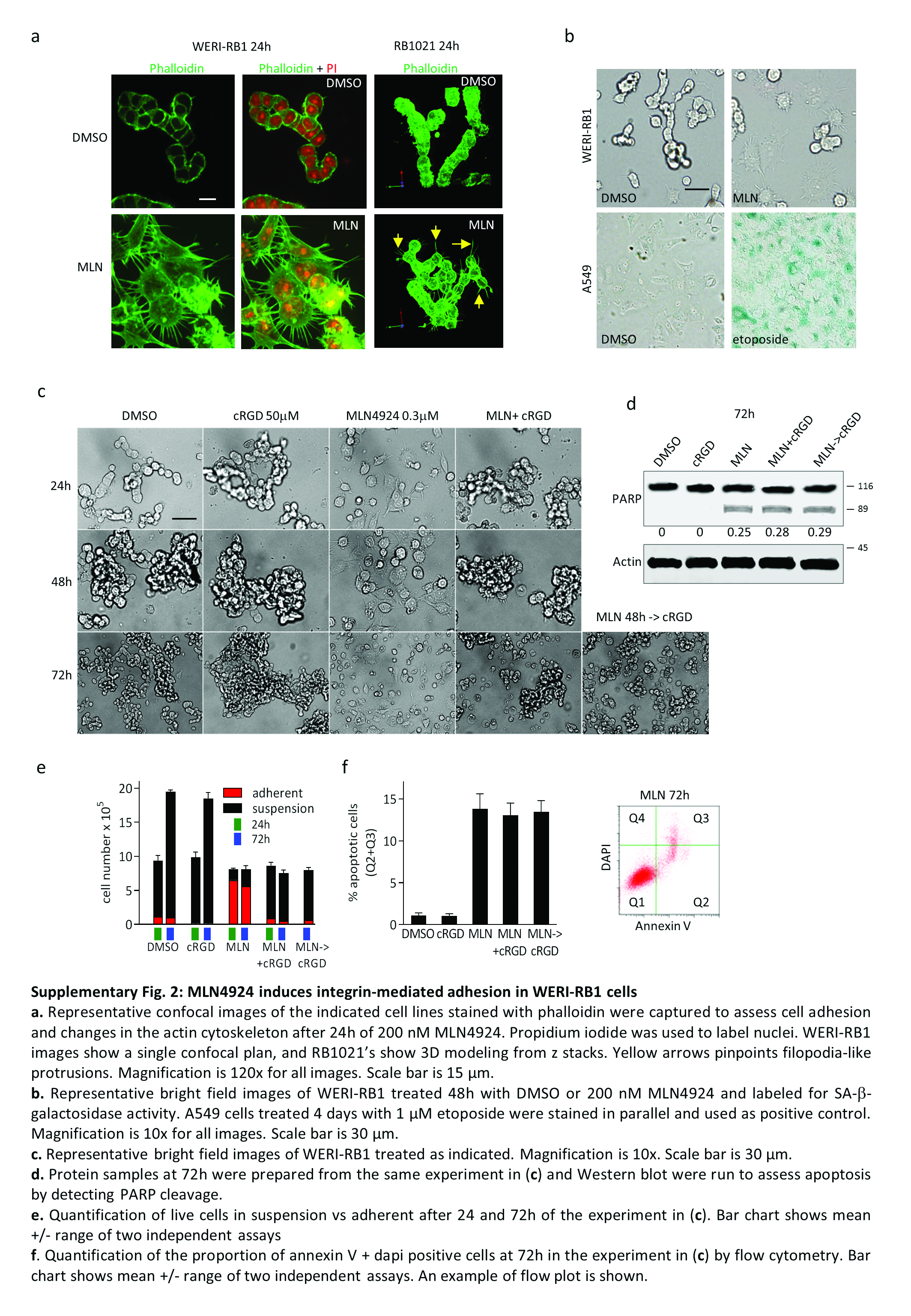

Supplement: Supplementary file 3 — Supplementary Figure 2 [file 41420_2020_237_MOESM3_ESM.tif]

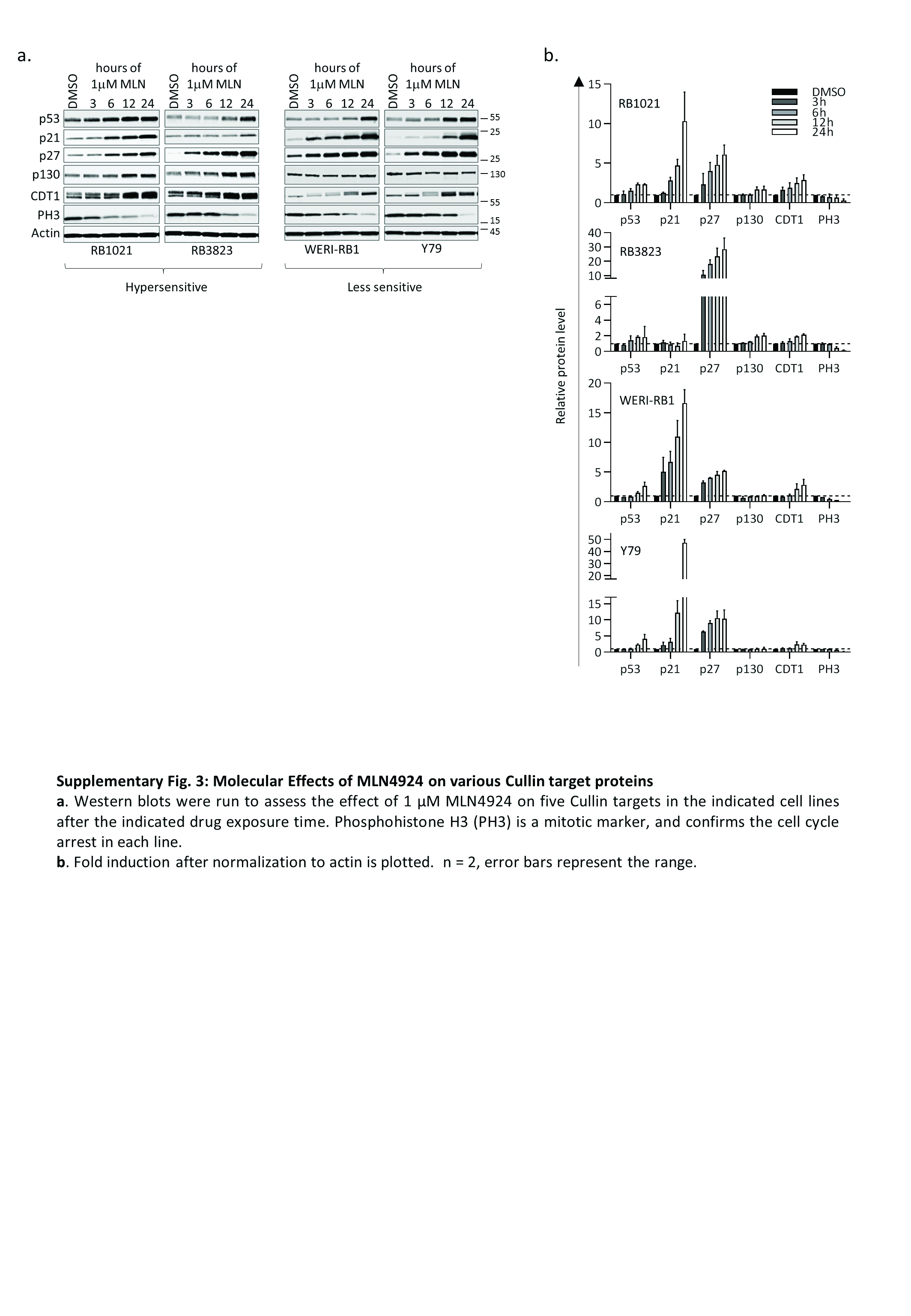

Supplement: Supplementary file 4 — Supplementary Figure 3 [file 41420_2020_237_MOESM4_ESM.tif]

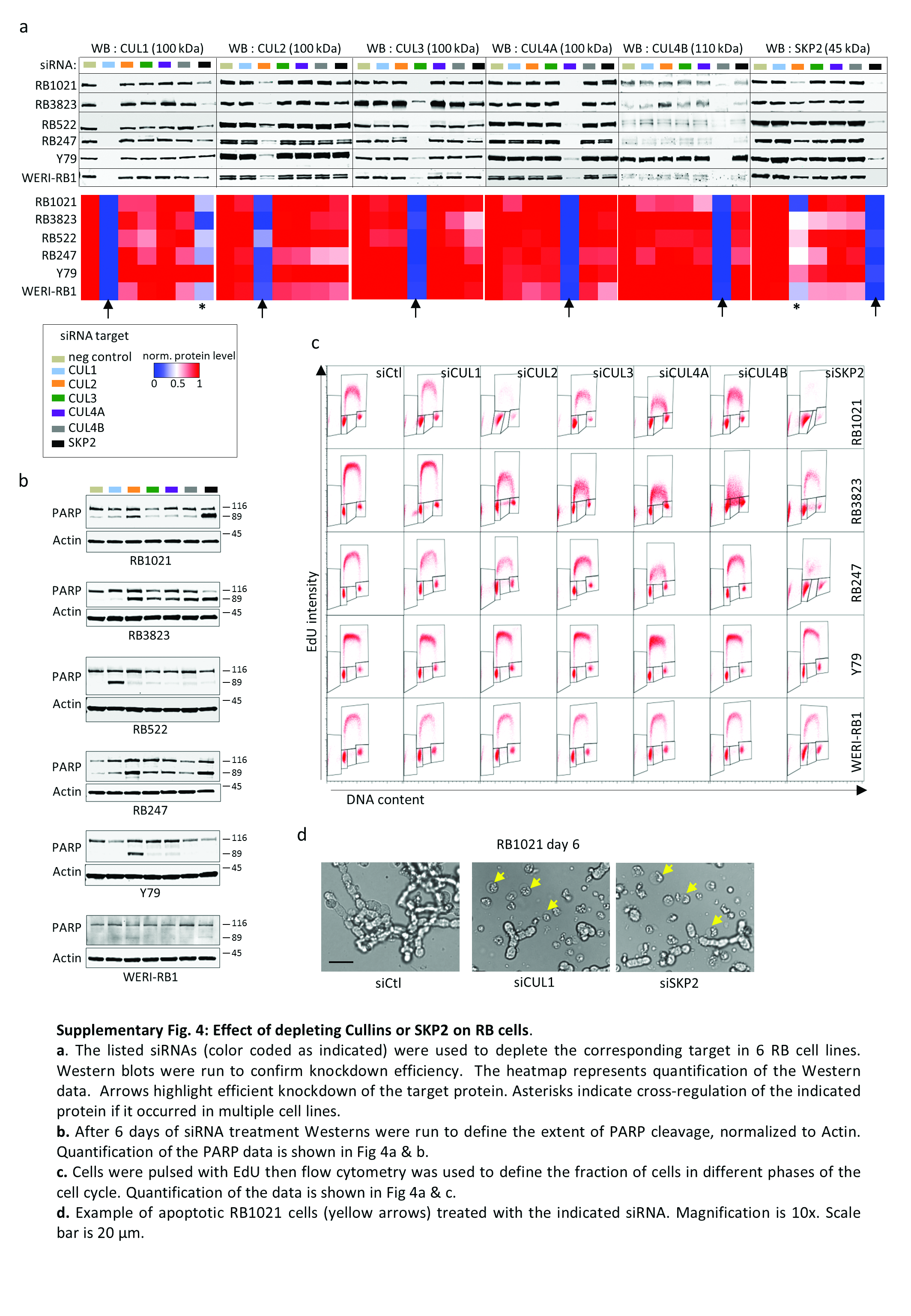

Supplement: Supplementary file 5 — Supplementary Figure 4 [file 41420_2020_237_MOESM5_ESM.tif]
