# Supplementary material for: Preclinical studies reveal MLN4924 is a promising new retinoblastoma therapy
Source: Cell Death Discov. 2020 Jan 20;6:2. doi: 10.1038/s41420-020-0237-8 (PMC7026052; doi:10.1038/s41420-020-0237-8)
Supplement: Supplementary file 6 — Supplementary table 1 [file 41420_2020_237_MOESM6_ESM.pdf]

Supplementary table 1. List of antibodies used in the study

Protein Target	MW kDa	Antibody dilution	Antibody Species	Company	Cat #	Buffer	application
beta-actin	42	1/5000	mouse	Sigma	5441	1x TBS 3% milk 0.05% Tween20	western blot
CDT1	65	1/1000	rabbit	Cell Signaling	#3386	1x TBS 5% BSA 0.05% Tween20	western blot
Cleaved PARP	89	1/1000	rabbit	Cell Signaling	5625S	1x TBS 3% milk 0.05% Tween20	western blot
CUL1	90	1/500	mouse	Santa Cruz	sc-17775	1x TBS 3% milk 0.05% Tween20	western blot
CUL2	80-90	1/500	mouse	Santa Cruz	sc-166506	1x TBS 3% milk 0.05% Tween20	western blot
CUL3	89	1/1000	rabbit	Bethyl Laboratories	A301-109A	1x TBS 5% BSA 0.05% Tween20	western blot
CUL4A	80, 82	1/1000	rabbit	Cell Signaling	2699	1x TBS 5% BSA 0.05% Tween20	western blot
CUL4B	104	1/1000	rabbit	Bethyl Laboratories	A303-863A	1x TBS 5% BSA 0.05% Tween20	western blot
MYCN	67	1/500	mouse	Santa Cruz	sc-53993	1x TBS 3% milk 0.05% Tween20	western blot
NEDD8	9	1/1000	rabbit	Cell Signaling	2745	1x TBS 5% BSA 0.05% Tween20	western blot
p130	130	1/1000	rabbit	Santa Cruz	sc-317	1x TBS 3% milk 0.05% Tween20	western blot
p21	21	1/1000	mouse	Santa Cruz	sc-6246	1x TBS 3% milk 0.05% Tween20	western blot
p21	21	1/1000	rabbit	Santa Cruz	sc-471	1x TBS 3% milk 0.05% Tween20	western blot
p27	24	1/1000	rabbit	Santa Cruz	sc-528	1x TBS 3% milk 0.05% Tween20	western blot
p53	53	1/1000	rabbit	Santa Cruz	sc-6243	1x TBS 3% milk 0.05% Tween20	western blot
PARP	116, 89	1/1000	rabbit	Cell Signaling	9532S	1x TBS 3% milk 0.05% Tween20	western blot
PH3	17	1/1000	rabbit	Millipore	06-570	1x TBS 3% milk 0.05% Tween20	western blot
pRB	105	1/1000	mouse	BD Biosciences	554136	1x TBS 3% milk 0.05% Tween20	western blot
SKP2	45	1/1000	rabbit	Santa Cruz	sc-7164	1x TBS 3% milk 0.05% Tween20	western blot
Cleaved Caspase 3 (AC3)		1/200	rabbit	Cell Signaling	9664	PBS 5% DS 0.1% Tween20	immunofluorescence
intact mitochondria		1/500	mouse	Millipore	MAB1273	PBS 5% DS 0.1% Tween20	immunofluorescence

TBS: Tris Buffered Saline
PBS: Phosphate Buffered Saline
BSA: Bovine Serum Albumine
DS: Donkey Serum

Probe	dilution	Company	Cat #	Buffer	Application
Alexa 488 Phalloidin	1/500	Life Technologies	A13279	PBS 3% BSA 0.5% TritonX	Immunofluorescence
