# Supplementary material for: Preclinical studies reveal MLN4924 is a promising new retinoblastoma therapy
Source: Cell Death Discov. 2020 Jan 20;6:2. doi: 10.1038/s41420-020-0237-8 (PMC7026052; doi:10.1038/s41420-020-0237-8)
Supplement: Supplementary file 7 — authors contribution form [file 41420_2020_237_MOESM7_ESM.pdf]

DECLARATION OF CONTRIBUTIONS TO ARTICLE

ADMC

Manuscript Number:

Journal Name: *Cell Death & Disease* (the 'Journal')

Proposed Title of the Contribution: Preclinical Studies Reveal MLN4924 is a Promising New Retinoblastoma Therapy (the 'Contribution)

Author(s): Arthur Aubry, Tao Yu, Rod Bremner (the 'Authors')

For all *CDDis* articles, each person named as an author in the published version must be able to show he or she has contributed substantially to the article.

Authorship credit should be based on 1) substantial contributions to conception and design, acquisition of data, or analysis and interpretation of data; 2) drafting the article or revising it critically for important intellectual content; and 3) final approval of the version to be published. Authors should meet conditions 1, 2 and 3.

Any person who cannot be shown to have made a substantial contribution to the article cannot be listed as an author in the final version. The name of any person who is deemed to have made a minor contribution can, however, appear in the Acknowledgments section of the article.

Please complete the table below to indicate the contributions of all named authors to the manuscript.

Author Full Name:	Specification of Contribution to the Manuscript:
Arthur Aubry	1,2,3
Tao Yu	1,2,3
Rod Bremner	1,2,3

Please complete the table below to indicate the contributions of all named authors to the figures.

Figure 1:

Arthur Aubry: experiment design, data acquisition, analysis and interpretation
Tao Yu: n/a
Rod Bremner: experiment design, data analysis and interpretation

Figure 2:

Arthur Aubry: experiment design, data acquisition, analysis and interpretation
Tao Yu: n/a
Rod Bremner: experiment design, data analysis and interpretation

Figure 3:

Arthur Aubry: experiment design, data acquisition, analysis and interpretation
Tao Yu: n/a
Rod Bremner: experiment design, data analysis and interpretation

Figure 4:

Arthur Aubry: experiment design, data acquisition, analysis and interpretation
Tao Yu: experiment design, data acquisition, analysis and interpretation
Rod Bremner: experiment design, data analysis and interpretation

Figure 5:

Figure 6:

Signed for and on behalf of the Author(s):	Print Name:	Date:
[signature]	Rod Bremner	4th Oct 2019
